# Unraveling the multiple interactions between phages, microbes and flavor in the fermentation of strong-flavor Baijiu

**DOI:** 10.1186/s40643-025-00852-1

**Published:** 2025-03-05

**Authors:** Huadong Zhang, Hongxia Zhang, Hai Du, Yan Zhang, Menghui Zhang, Xiaowei Yu, Yan Xu

**Affiliations:** 1 Laboratory of Brewing Microbiology and Applied Enzymology, The Key Laboratory of Industrial Biotechnology, Ministry of Education, State Key Laboratory of Food Science and Technology, School of Biotechnology, Jiangnan University, 1800 Lihu Ave, Wuxi, 214122 Jiangsu China; 2 College of Life Sciences, Shanxi Normal University, Taiyuan, 030000 Shanxi China; 3 Key Laboratory of Systems Biomedicine (Ministry of Education), Shanghai Center for Systems Biomedicine, Shanghai Jiao Tong University, Shanghai, China; 4 State Key Laboratory of Microbial Metabolism, Shanghai Jiao Tong University, Shanghai, China; 5 Joint International Research Laboratory of Metabolic and Developmental Sciences, School of Life Sciences and Biotechnology, Shanghai Jiao Tong University, Shanghai, China

**Keywords:** Baijiu, Virus, Bacteria, Fungal, Viral metagenomics, Fermented foods

## Abstract

The fermentation process of strong-flavor Baijiu represents a complex and unique ecosystem, characterized by the involvement of various microorganisms that drive intricate biochemical reactions, ultimately contributing to the distinct flavor profile of the Baijiu. Viruses may affect the succession of microorganisms and thus affect the style and quality of the product. However, the interaction between viruses and microorganisms during the fermentation of Baijiu is still unclear. Here we combined viral metagenomics and amplicon sequencing, physicochemical analysis, and GC–MS detection with temporal sampling to study the dynamics of viral and microbial communities, physicochemical properties, and flavor compounds during strong-flavor Baijiu fermentation. Viral metagenomic analysis revealed 513 viral operational taxonomic units (vOTUs), encompassing 34 viral families. Principal coordinates analysis (PCoA) demonstrated significant differences in vOTUs at different fermentation stages. Notably, the microbial community exhibited distinct succession patterns at various fermentation stages; it changed rapidly during the initial five days, with similarities observed between days 10 and 20. Volatile profile analysis identified 38 flavor components in fermented grains, comprising 16 ester compounds, 11 alcohols, and 8 acids, with the majority formed between days 10 and 30. The Spearman's rank correlation analysis revealed that Peduoviridae exhibited a negative correlation with *Gluconobacter*. Genomoviridae showed a negative correlation with *Issatchenkia*, *Penicillium*, and *Monascus*. These findings highlight the potential for complex interactions between viruses and microbial communities during Baijiu fermentation, underscoring the importance of considering viral communities in studies of the microbial ecology of fermented foods.

**Graphical Abstract:**

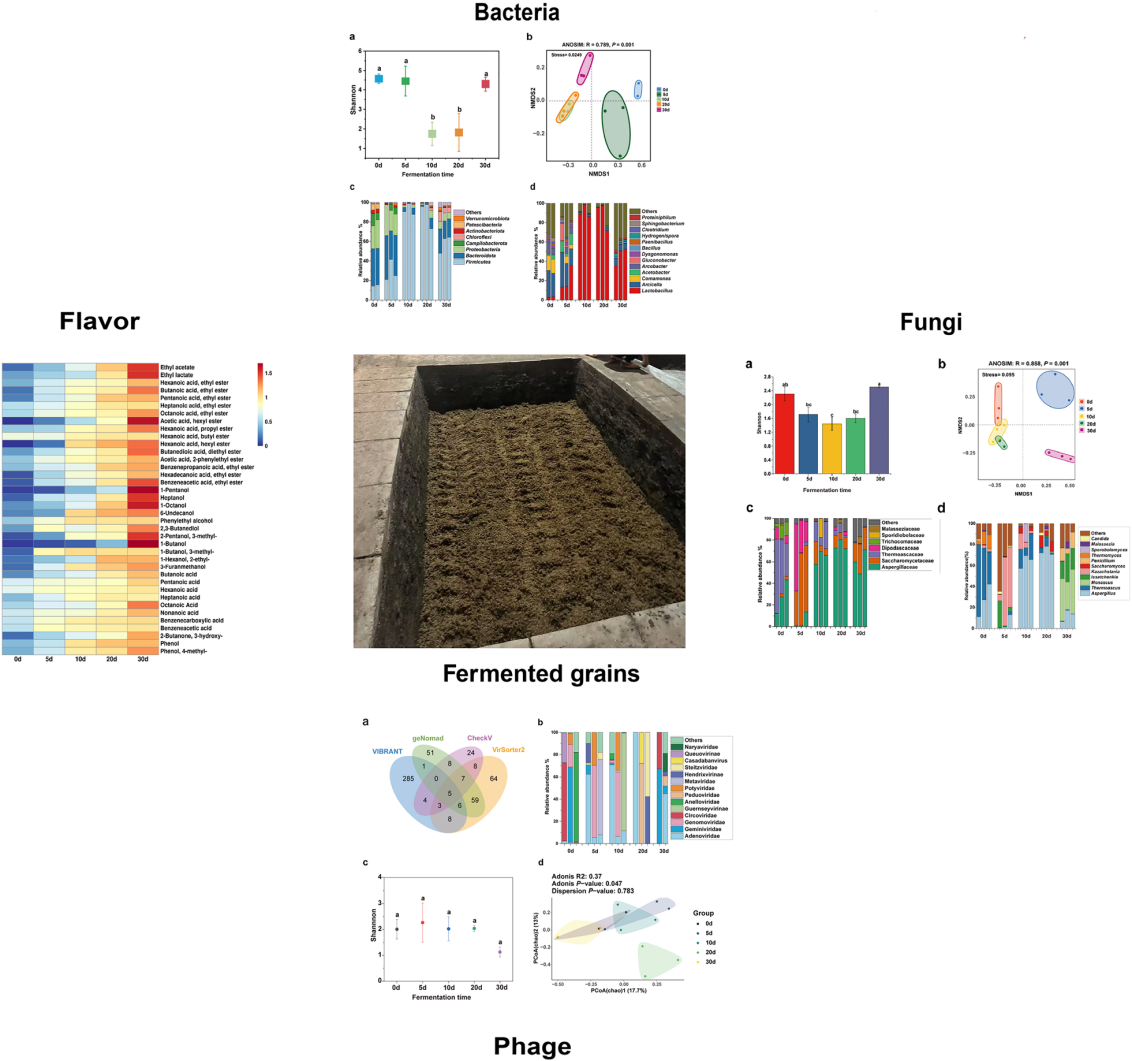

**Supplementary Information:**

The online version contains supplementary material available at 10.1186/s40643-025-00852-1.

## Introduction

Strong-flavor Baijiu accounts for approximately 50% of the Chinese Baijiu market and is widely loved by consumers for its unique flavor (Hong et al. 2023). The flavor profile of strong-flavor Baijiu is characterized by a prominent cellar aroma, mellow sweetness, harmonious fragrance, and a long aftertaste (Wang et al. 2022a, b; Zou et al. 2018). Ethyl caproate is the primary substance responsible for the main aroma of this type (He et al. 2020). Other esters, including ethyl acetate, ethyl lactate, ethyl butyrate, and ethyl valerate, are also present in higher concentrations (Qiu et al. 2024). Acids such as acetic acid, lactic acid, caproic acid, butyric acid, and caprylic acid contribute to the liquor’s taste and help reduce its irritation (He et al. 2021a, b). In addition to ethanol, strong-flavor Baijiu contains higher alcohols such as n-propanol, isobutanol, isopentanol, and phenylethanol, which cooperate with ester compounds to enhance the release of aroma (Wang et al. 2024). The unique aroma of strong-flavor Baijiu is closely linked to its distinctive production process, which includes the use of medium–high temperature Daqu as a saccharification and fermentation agent, the mixing of fermented grains with raw grains distilled to produce fresh Baijiu, and fermentation in mud cellars (Chai et al. 2024). These processes foster a rich and diverse microbial community (Huang et al. 2024). Understanding the dynamic changes of microbial communities and their interrelationships during the fermentation of strong-flavor Baijiu is crucial for revealing the mystery of its flavor formation, optimizing fermentation technology, and improving product quality.

In previous studies on microorganisms involved in Baijiu fermentation, researchers have primarily focused on two major microbial groups: bacteria and fungi (Liu et al. 2024; Tan et al. 2022). The succession and interactions of diverse functional microorganisms result in the development of its unique flavor (Liu and Sun 2018; Tan et al. 2019; Wu et al. 2021a, b). Filamentous fungi, represented by genera such as *Aspergillus* and *Rhizopus*, are capable of producing a range of enzymes, including glucoamylase, α-amylase, proteases, and lipases/esterases. These enzymes play a crucial role in the early stages of fermentation by breaking down complex macromolecules like starch and proteins into simpler compounds, such as glucose and amino acids (Wang et al. 2020; Wu et al. 2021a, b). Lactic acid bacteria (LAB) synthesizes flavor compounds and their precursors, such as lactic acid and acetic acid (Hu et al. 2022; Wang et al. 2022a, b). Both *Saccharomyces* and non-*Saccharomyces* species produce ethanol, ethyl acetate, phenylethyl alcohol and other flavor compounds (Hu et al. 2021; Yuan et al. 2023a, b; Zhang et al. 2022). Abundant volatile acids produced by microorganisms in pit mud can undergo esterification with ethanol generated by yeast in fermented grains, resulting in the formation of ester flavor compounds,such as ethyl caproate, ethyl lactate, ethyl butyrate (Gao et al. 2021; Zhang et al. 2020). However, within this complex microbial ecosystem, viruses—a unique and often overlooked biological factor—have not received adequate attention regarding their influence on microbial succession and fermentation processes. Their mechanisms of action and ecological roles in Baijiu fermentation remain largely unexplored and poorly understood.

With the advancement of metagenomics technology, the viral community in fermented foods can now be more comprehensively characterized. For instance, the prolonged and widespread presence of phages in cheese revealed the succession of phages infecting LAB at the early stages of cheese ripening, and ripening bacteria (Actinomycetota, Pseudomonadota in particular) in the later stages (Paillet et al. 2024). An 82-year metagenomic study of Swiss cheese starters revealed that two highly abundant *Streptococcus* phages coexist stably within the community, without negatively affecting bacterial growth or strain persistence. Instead, they may have conferred resistance to lytic phages through a double infection mechanism or provided the host with a growth advantage over competing sensitive strains (Somerville et al. 2022). Phage communities in kimchi seemed to be more geographically characterized than bacteria (Jung et al. 2018). The phage community structures in different types of Daqu were significantly different, with Siphoviridae being the main phage family in medium-temperature Daqu (Kang et al. 2022). Additionally, during the fermentation of sauce-flavor Baijiu, phage induction liquid was found to have a significant inhibitory effect on *Bacillus* (Du et al. 2022). However, viral community composition and function are still relatively unexplored during the fermentation process of strong-flavor Baijiu.

In this study, the dynamics of viral and microbial community, physicochemical properties, flavor substances in fermented grains across different fermentation time points (0-, 5-, 10-, 20-, and 30-day) during the strong-flavor Baijiu fermentation process, were analyzed by using a combination of viral metagenomics, third-generation amplicon full-length sequencing, physicochemical analysis and GC–MS detection methods. And we calculated the relationship between viruses, microorganisms, and flavors. Furthermore, the host of the virus was predicted, and the functional genes of the virus were annotated. This study aims to: Address the gap in the study of viral diversity in fermented foods by expanding our knowledge of viral communities within the Baijiu fermentation ecosystem, thereby providing essential data for future research. Uncover the potential relationship between viruses and microorganisms.

## Materials and methods

### Sample collection

Fermented samples were collected from a famous Baijiu factory in Sichuan Province, China, in 2022. Based on the results of a previous study (Yuan et al. 2023a, b), 5 sampling time points (days 0, 5, 10, 20, and 30) were set to collect fermented grains. Samples were collected during the first 30 days of fermentation, as this period represents the peak of microbial activity and the completion of alcohol fermentation (Hu et al. 2021). Beyond this stage, microbial succession stabilizes, and pit mud microorganisms continue to produce short- and medium-chain fatty acids, which contribute to ester formation (Gao et al. 2021). The fermentation cellar for the strong-flavor Baijiu is approximately 2 m deep and utilizes a solid-state fermentation process. To ensure that the sampling time points accurately represent the fermentation conditions throughout the entire cellar, three samples (100 g each) were taken from three layers (depths of 0.5 m, 1 m, and 1.5 m) at each fermentation time point (Fig S1) (Du et al. 2022). A total of 15 fermented grain samples were collected and flash frozen with liquid nitrogen stored in the lab at – 80 ℃ until analysis.

### Determining physicochemical properties

The temperature of the sampling depth was measured by thermometer before sample collection. For moisture detection, 10 g of fermented grain was dried at 105 ℃ for 3 h, and weight loss was then measured. A pH meter (Delta320, Mettler Toledo, Switzerland) was utilized to measure the pH by direct insertion into the sample suspension (10 g/100 mL). To analyze the ethanol, glucose, and organic acid content in fermented grain samples, 5 g of each sample was mixed with 30 mL of distilled water and subjected to ultrasonic treatment at 0 ℃ for 30 min. The mixture was then centrifuged at 4 ℃ and 1000 × *g* for 10 min. The resulting supernatant was filtered through a 0.22 μm syringe filter (Nylon Acrodisc, Waters Co., Milford, MA) before analysis (Wang et al. 2018). The compound content was determined using HPLC (Agilent) with an Aminex HPX-87H column (Bio-Rad). The column was eluted at 60 ℃ with a degassed mobile phase of 5 mM H_2_SO_4_ at a flow rate of 0.6 mL/min. All compounds were detected with a refractive index detector (RID) at 40 ℃, with an injection volume of 10 μL and a total run time of 25 min.

### Volatile compound analysis

Volatile compounds in fermented grains were analyzed using headspace solid-phase microextraction coupled with gas chromatography-mass spectrometry (HS–SPME–GC–MS). A sample of 5 g of fermented grains was mixed with 30 mL of distilled water and subjected to ultrasonic treatment at 0 ℃ for 30 min. The resulting mixture was then centrifuged at 1000 × *g* for 10 min at 4 ℃. Subsequently, 5 mL of the supernatant was transferred to a headspace vial containing 2.5 g of sodium chloride and 8 μL of menthol as an internal standard (106.25 mg/L).

Volatile aroma compounds were extracted using an automatic headspace sampling system (MultiPurposeSample MPS 2 with SPME adapter, Gerstel Inc., Mülheim an der Ruhr, Germany) equipped with a 50/30 μm divinylbenzene/carboxen/poly(dimethylsiloxane) (DVB/CAR/PDMS) fiber (2 cm, Supelco Inc., Bellefonte, PA, USA). The tightly capped sample vials were equilibrated at 50 ℃ for 5 min before extraction, which was conducted for 45 min at the same temperature under stirring at a rotation speed of 250 × g. After extraction, the SPME fiber was introduced into the GC injection port for desorption at 250 ℃ for 5 min (Gao et al. 2014).

The gas chromatography–mass spectrometry analysis was performed using an Agilent 6890 GC coupled with an Agilent 5975 N mass selective detector (MSD). Samples were analyzed on a DB-Wax column (30 m × 0.32 mm i.d. × 0.25 μm, J&W Scientific). Helium was employed as the carrier gas at a constant flow rate of 1 mL/min. The oven temperature program was as follows: initial temperature of 50 ℃ held for 2 min, followed by a ramp to 230 ℃ at a rate of 4 ℃/min, where it was held for an additional 15 min. The mass spectrometer operated in electron ionization (EI) mode at 70 eV, with the ion source temperature set to 230 ℃. Full-scan acquisition was conducted over a mass range of 35–550 amu to characterize the compounds, which were identified by comparison with reference spectra from the NIST 11 library (Agilent Technologies, Inc.) (Hu et al. 2021).

### Viral DNA extraction, sequencing and assembly

For viral DNA extraction, virus-like particles (VLP) in the fermented grain were first purified and concentrated by filtration and centrifugation following the previously described protocol (Du et al. 2022; Kang et al. 2022). 5 g of fermented grain was taken for grinding and added to 5 volumes of precooled sterile Stabilization Buffer (SB) (0.2 M NaCl, 5 mM CaCl_2_, 50 mM Tris–HCl, 5 mM MgCl_2_, pH 7.5). The mixture was vortexed for 5 min. After three rounds of freeze-thawing, the sample was centrifuged at 12,000 × *g* for 5 min to remove the precipitate. The cell fragments were removed using 0.45 μm and 0.22 μm filter membranes. The supernatant was transferred via syringe to an ultracentrifugation tube containing 28% (w/w) sucrose. After careful trimming, the sample was centrifuged at 160,000 × *g* for 2 h at 4 ℃ using a HIMAC CP 100wx ultracentrifuge (Hitachi, Tokyo, Japan). Then the precipitate was suspended in 200 µL of SB buffer after the supernatant was removed. An enzyme mixture solution (720 μL of ddH_2_O, 90 μL 10 × DNase I buffer, 90 μL 1 U/μL DNase I, 0.9 μL 100 mg/mL RNase A) was added to the suspension and incubated for 60 min at 37 ℃. The suspension was incubated at 65–75 ℃ for 10 min to inactivate the enzyme reaction before being centrifuged at 2000 × *g* for 5 min. The supernatant was kept at – 20 ℃ for future viral genomic studies. DNA extraction from the VLP fraction was performed using the TaKaRa MiniBEST Viral DNA Extraction Kit Ver.5.0 and amplified using the Qiagen REPLI-g Cell WGA & WTA Kit (150,054). Finally, the amplification products were quantified using Thermo NanoDrop One, Life Technologies Qubit 4.0, and 1% agarose gel electrophoresis.

Sequencing libraries were generated using ALFA-SEQ DNA Library Prep Kit for Illumina (FINDROP, Guangzhou) following manufacturer’s recommendations index and barcodes were added. The DNA concentration was quantified using the Qubit^®^ dsDNA HS Assay Kit (Life Technologies, Grand Island, NY), and the library quality was assessed using the Bioptic Qsep400 system. At last, the library was sequenced on an Illumina Novaseq 6000 and 150 bp paired-end reads were generated (Liu et al. 2023).

The raw Illumina reads were filtered by Trimmomatic (v0.36). Afterwards, the clean reads were assembled using Megahit (v1.1.2) with parameters as: k—min 35, k—max 95, k—step 20 and – presets meta—large, – min—contig—len = 300 for viruses (Xia et al. 2023).

### Viral contig identification for taxonomic assignment and functional annotation

To enhance the accuracy of viral sequence identification, contigs longer than 2 kb were screened using VirSorter2 (v2.2.4, default parameters) geNomad (v1.8.0, default parameters) and VIBRANT (v1.2.1, default parameters). Viral contigs identified by both tools were quality-controlled using CheckV (v1.0.1, default parameters). To be declared as viral, a contig had to meet at least one of the following criteria: declared “complete,” “high,” or “medium” quality by either VIBRANT or CheckV, declared score > 0.95 for VirSorter2 and score > 0.9 for geNomad (Camargo et al. 2023; Guo et al. 2021; Kieft et al. 2020; Nayfach et al. 2020). Viral contigs were clustered into viral OTUs (vOTUs) based on ≥ 95% nucleotide identity over ≥ 85% coverage of the shorter sequence using the CD-HIT (v4.8.1) (Fu et al. 2012). The longest sequence of the vOTU cluster was retained as the representative sequence. Viral OTU abundance (RPKM, reads per kilobase per million) was calculated using CoverM (v0.6.1) (https://github.com/wwood/CoverM) with the parameters: “–contig –min-read-aligned-percent 75 –min-read-percent-identity 95 –min-covered-fraction 75 –contig-end-exclusion 0 -m rpkm.” Prodigal (v2.6.3) was used to predict the open reading frames (ORFs) of vOTUs (Hyatt et al. 2010). EggNOG-mapper was used to annotate the predicted ORFs functional information (Cantalapiedra et al. 2021). For viral classification standards, first we annotate the viruses using PhaGCN2 (v2.1) with default options (Jiang et al. 2023). The remaining unclassified vOTUs were annotated by geNomad (v1.8.0) with default options (Camargo et al. 2023).

### Microbial DNA extraction and sequencing

E.Z.N.A. Soil DNA Kit (Omega Biotek, Norcross, GA, USA) was used to extract the total microbial DNA from all samples following manufacturer’s instructions (Wu et al. 2024). Extracted DNA samples were sent to Guangdong Meige Gene Technology Co., LTD. (Guangdong, China) for high throughput sequencing. The V1-V9 hypervariable region of 16S rRNA was amplified with the primers of 27F and 1492R (Okazaki et al. 2021), while the internal transcribed spacer region (ITS1-4) was amplified with ITS1F and ITS4R (Furneaux et al. 2021). Sequencing was performed on PacBio Sequel II using equimolar amounts of PCR products. Clean reads were obtained by processing the raw data using SMRT Link (v6.0). High-quality segments were clustered for generating OTUs with a 97% identity threshold by using USEARCH (V10, http://www.drive5.com/usearch/). Representative sequence species annotation information for OTUs was obtained using RDP (16S), Silva (16S) and UNITE (ITS) database comparisons (Ban et al. 2024).

### Phage-bacterium interaction analysis

The host of the phage was further predicted using iPHoP (v1.3.3) with default parameters (Arumugam et al. 2023), and the highest-scoring candidates were selected as potential hosts. Deephage (v1.0) was used to predict the lifestyles of phages with default options (Wu et al. 2021a, b).

### Statistical analysis

The α-diversity and β-diversity of the samples were analyzed using the “vegan” package in R software (v 4.1.1). Non-metric multidimensional scaling analysis (NMDS) using Bray–Curtis distances was performed to assess community dissimilarities. The significance of differences in parameters were compared using t-test in SPSS 26.0. The relationships between dominant genera and physicochemical properties were analyzed using a correlation heatmap generated by the “pheatmap” package in R. The association between microbial communities and physicochemical properties was assessed through a Mantel test implemented via the “ggcor” package. To evaluate the Spearman’s rank correlation coefficient between dominant genera and differential compounds, the “Hmisc” package was utilized. A co-occurrence network was then constructed based on criteria of |ρ|> 0.7 and *p* < 0.05, and visualized using Gephi software (v.0.9.2).

## Results

### Dynamics of fermentation parameters and volatiles during fermentation

**Fig. 1:**
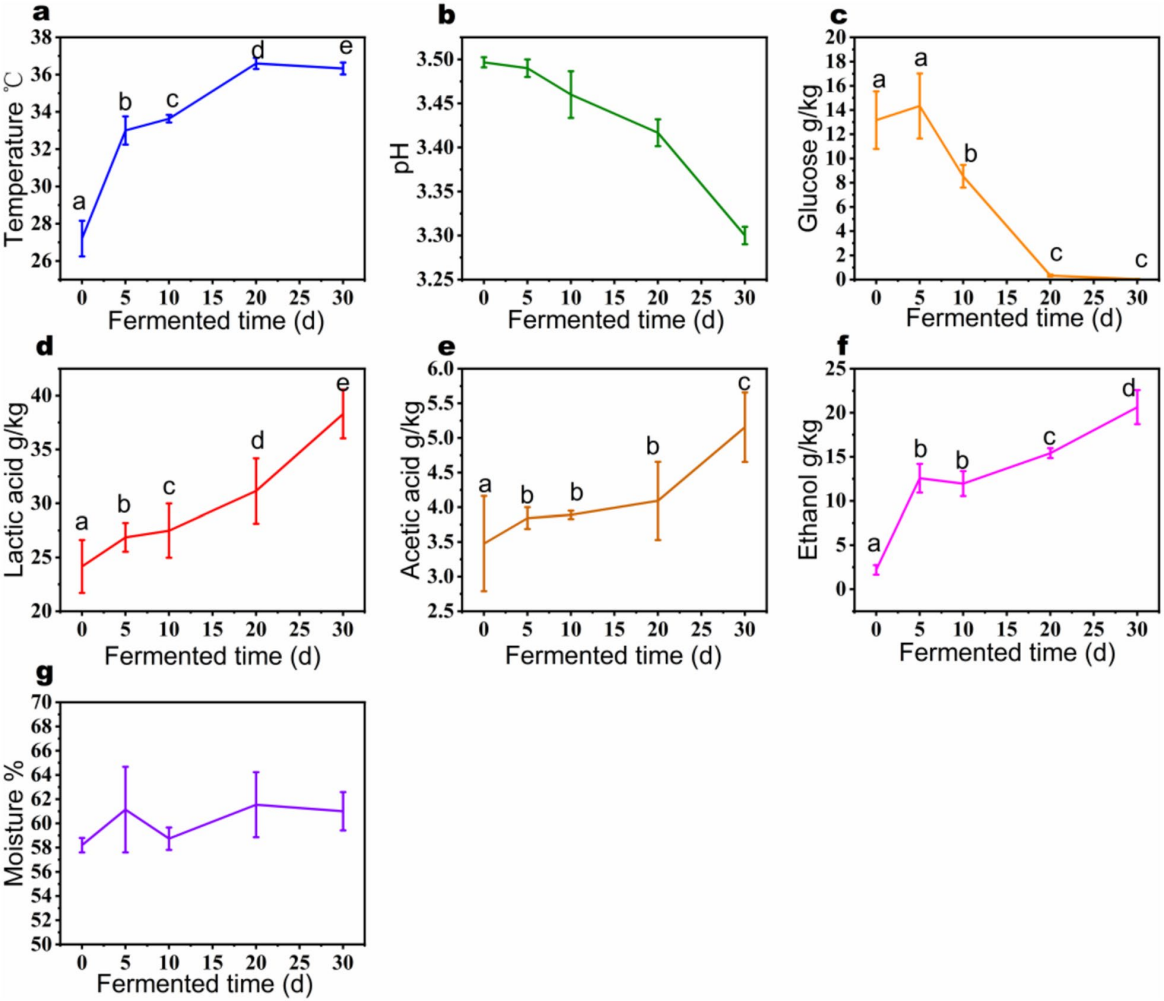
Dynamics of fermentation parameters in strong-flavor Baijiu fermentation process. **a** Temperature. **b** pH. **c** Glucose. **d** Lactic acid. **e** Acetic acid. **f** Ethanol. **g** Moisture. The data showed the average and standard deviation of 3 depth samples at the same fermentation time. Different letters on each picture represent significant differences, *P* < 0.05. Same letters and no letters represent no significant differences

During the fermentation process, the temperature gradually rose from 27.2 ± 0.95 ℃ to 36.3 ± 0.32 ℃ (Fig. 1a). The pH slowly decreased from 3.5 ± 0.01 to 3.3 ± 0.01 (Fig S3b), the glucose decreased from 13.15 ± 2.38 g/kg to 0.03 ± 0.02 g/kg (Fig. 1c). The content of lactic acid, acetic acid, and ethanol all increased (Fig. 1d, e, f), with the highest rate of ethanol production observed from day 0 to day 5. The moisture content did not change significantly during the fermentation process from day 0 to day 30 (Fig. 1g).

**Fig. 2:**
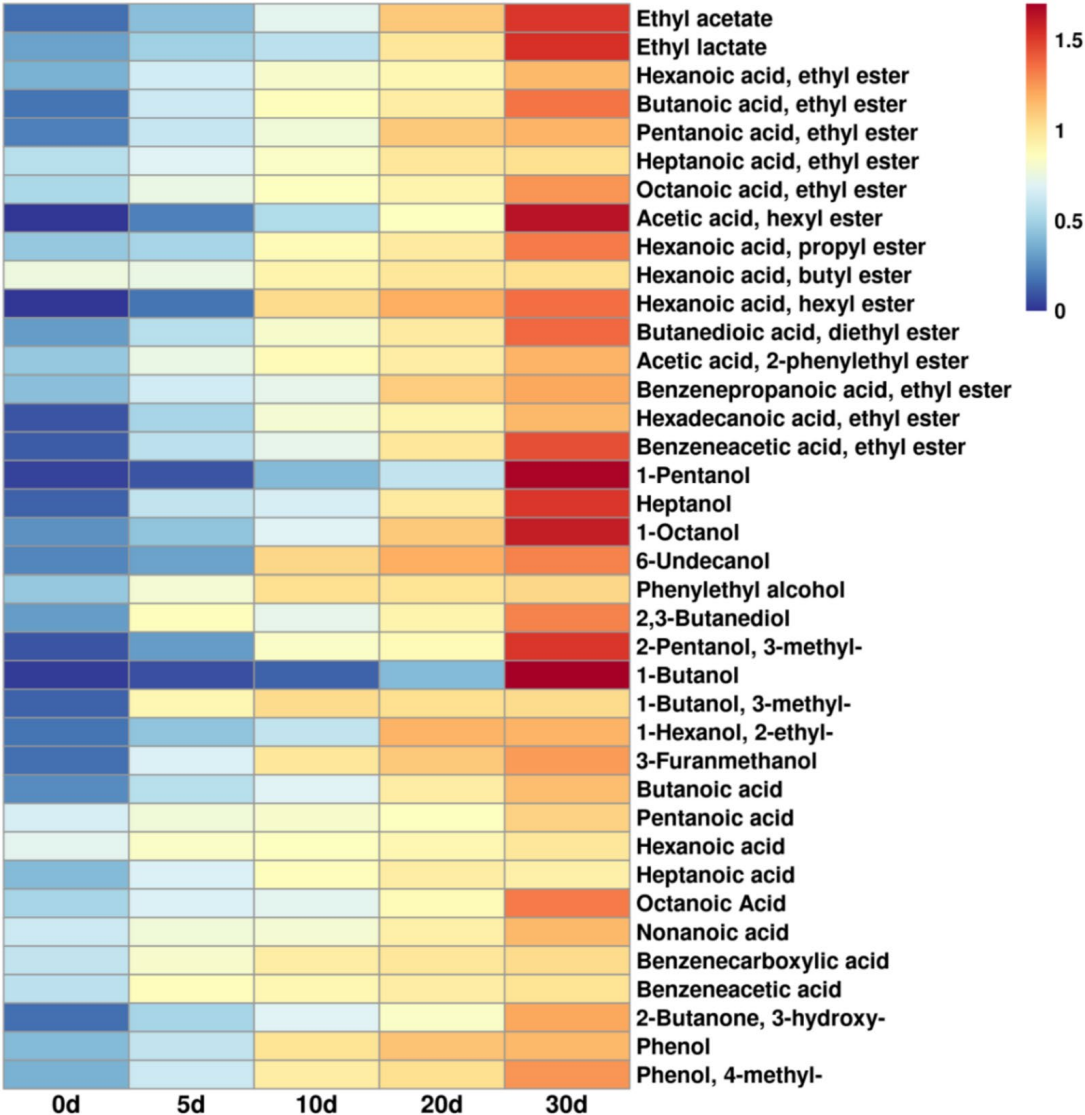
The comparison of flavour compounds in fermented grains. The relative values for each compound in different samples were calculated based on the normalized peak areas from HS–SPME–GC–MS analysis. The data showed the average of 3 depth samples at the same fermentation time

We conducted a semi-quantitative analysis of 38 flavor components in fermented grains, including 16 ester compounds, 11 alcohol compounds, and 8 acid compounds (Fig. 2). During the fermentation process, the content of ester species increased significantly from day 10, reaching the highest concentration of all ester compounds on day 30. The distinctive feature of Baijiu, compared to other distilled spirits, lies in the variety and richness of ester substances, which is mainly related to the metabolism of various microorganisms during fermentation. Ethyl acetate, ethyl lactate, ethyl hexanoate, and ethyl butyrate are the four highest-content esters in strong-flavor Baijiu (Yuan et al. 2023a, b).

During the Baijiu fermentation process, a considerable amount of higher alcohols are produced, such as 1-pentanol, 1-octanol, 6-undecanol, phenylethyl alcohol, 3-methyl-2-pentanol, 1-butanol, 3-methyl-1-butanol, 2-ethyl-1-hexanol. These higher alcohols contribute to the diverse aroma of the liquor; for example, phenylethyl alcohol has a rose-like scent, while 3-methyl-1-butanol has a nail polish-like smell (Zhang et al. 2021). The production rates of these higher alcohols vary during fermentation, with 1-pentanol and 1-butanol being produced significantly between days 20 to 30.

The period from day 10 to day 20 saw a higher production rate of butanoic acid, while pentanoic acid and hexanoic acid began to increase slowly from day 5. Acid compounds serve as precursors to the formation of many esters in Baijiu, such as hexanoic acid and ethanol esterifying to form ethyl hexanoate, and butanoic acid and ethanol esterifying to form ethyl butyrate. The appropriate amount of acids in Baijiu can reduce irritation and enhance the release of aroma (He et al. 2021a, b).

### Bacterial community composition during strong-flavor Baijiu fermentation

**Fig. 3:**
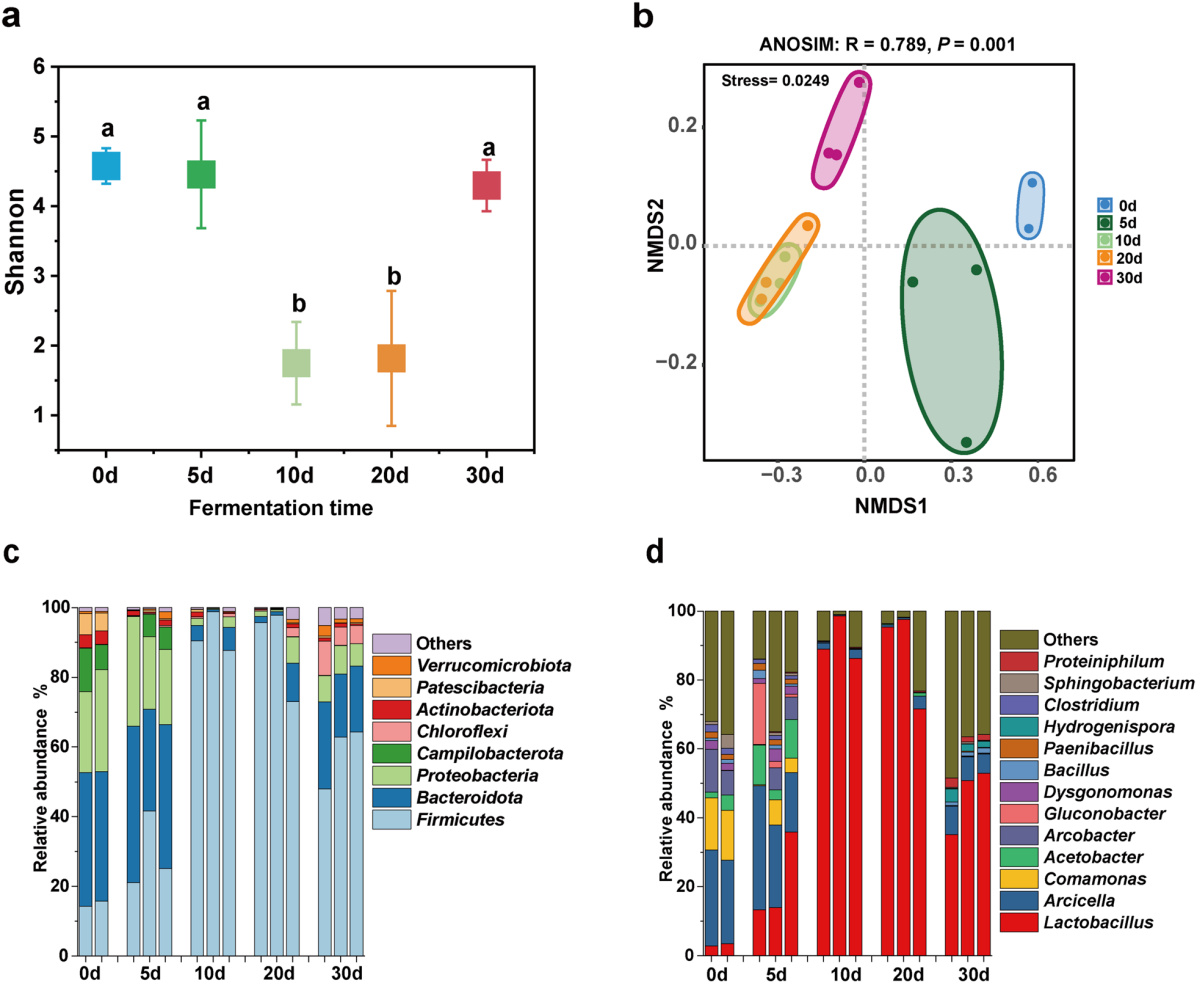
Bacterial succession pattern during strong-flavor Baijiu fermentation. **a** Bacterial Shannon index, the same letter represents no significant difference, and different letters represent significant differences. Data are presented as the means 3 standard deviations (by Tukey’s test, *P* < 0.05). **b** Bacterial NMDS analysis, ANOSIM: R = 0.789, *P* = 0.001. **c** Phyla detected at a relative abundance of ≥ 0.1% in this study. **d** Genera detected at a relative abundance of ≥ 1% in this study. The sample from day 0 at 1 m failed to be sequenced due to issues with library construction

The bacterial succession pattern during the first 30 days of fermentation was explored using third-generation amplicon full-length sequencing (Fig. 3). However, the sample from day 0 at 1 m failed to be sequenced due to issues with library construction. The results revealed that the Shannon index of the bacterial community at days 0, 5 and 30 of fermentation was significantly higher than at days 10 and 20, indicating notable differences in species diversity across different fermentation periods (Fig. 3a). We also used NMDS to study the Bray–Curtis distance of bacterial taxa composition among different fermentation days. The bacterial taxonomic composition clearly differed between the fermentation days (the Adonis analysis, *P* < 0.05), but not between days 10 and 20 (Fig. 3b). We found that Firmicutes, Proteobacteria, and Bacteroidota accounted for over 80% of the bacterial abundance during the first 5 days. The abundance of Proteobacteria and Bacteroidota decreased at days 10 and 20, and increased at day 30 (Fig. 3c). As shown in Fig. 3d, the bacterial population structure was diverse during the first 5 days of fermentation, with significant variation within the group at day 5. There were 11 bacterial genera with a relative abundance greater than 1%, and the relative abundance of *Lactobacillus* was only 1.1% on day 0. However, *Lactobacillus spp.* became the dominant genus on days 10 and 20 of fermentation, with its relative abundance exceeding 80%.

### Fungal community composition during strong-flavor Baijiu fermentation

**Fig. 4:**
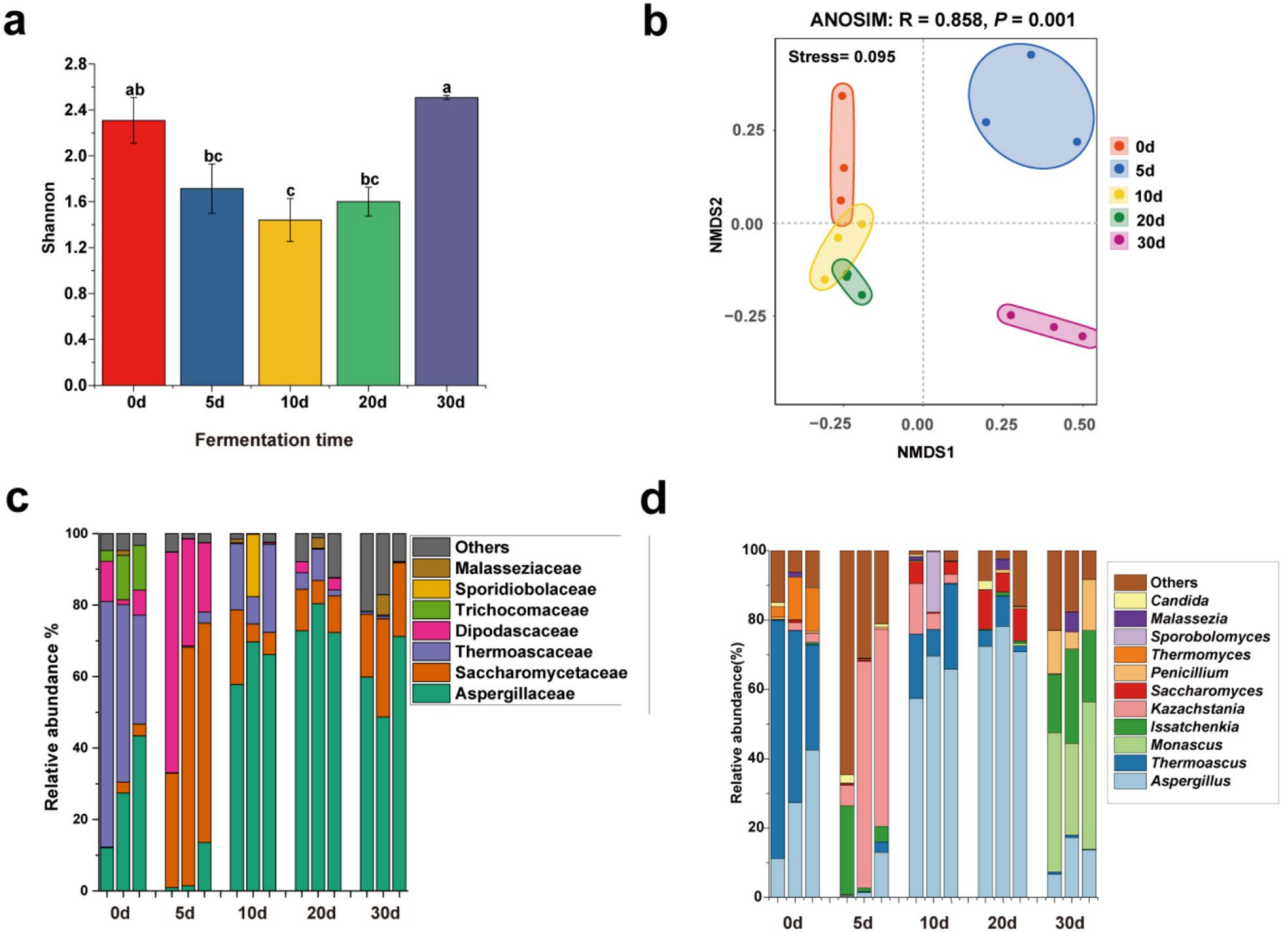
Fungi succession pattern during fermentation of strong-flavor Baijiu. **a** Fungi Shannon index, the same letter represents no significant difference, and different letters represent significant differences. **b** Fungi NMDS analysis, ANOSIM: R = 0.858, *P* = 0.001. **c** Family detected at a relative abundance of ≥ 0.1% in this study. **d** Genera detected at a relative abundance of ≥ 1% in this study

The diversity and composition of fungal communities in fermentation samples were investigated using third-generation full-length amplicon sequencing, as shown in Fig. 4. The alpha diversity of fungal communities exhibited a decreasing and then increasing trend (Fig. 4a). NMDS results based on the Bray–Curtis distance indicated significant differences in fungal community structure across different fermentation days (Adonis analysis, *P* < 0.05) (Fig. 4b). At the beginning of fermentation, the dominant fungi were primarily from the Thermoascaceae family, which exhibited a relative abundance of over 50%. By the fifth day of fermentation, the Saccharomycetaceae family emerged as the dominant group, with yeast populations increasing significantly, corresponding to a rise in the ethanol production rate. After 10 days of fermentation, Aspergillaceae became the dominant family, with a relative abundance exceeding 50% in each sample, followed by Saccharomycetaceae (Fig. 4c).

The fungal community structure at the genus level during fermentation was illustrated in Fig. 4d. *Aspergillus*, *Thermoascus*, and *Thermomyces* were the dominant genera, comprising over 70% of the relative abundance on day 0 of fermentation. *Issatchenkia* and *Kazachstania* became the dominant genera on day 5. The fungal community structure was similar on days 10 and 20, while *Monascus* and *Issatchenkia* dominated on day 30.

### Viral taxonomy classification and diversity in strong-flavor Baijiu fermentation

**Fig. 5:**
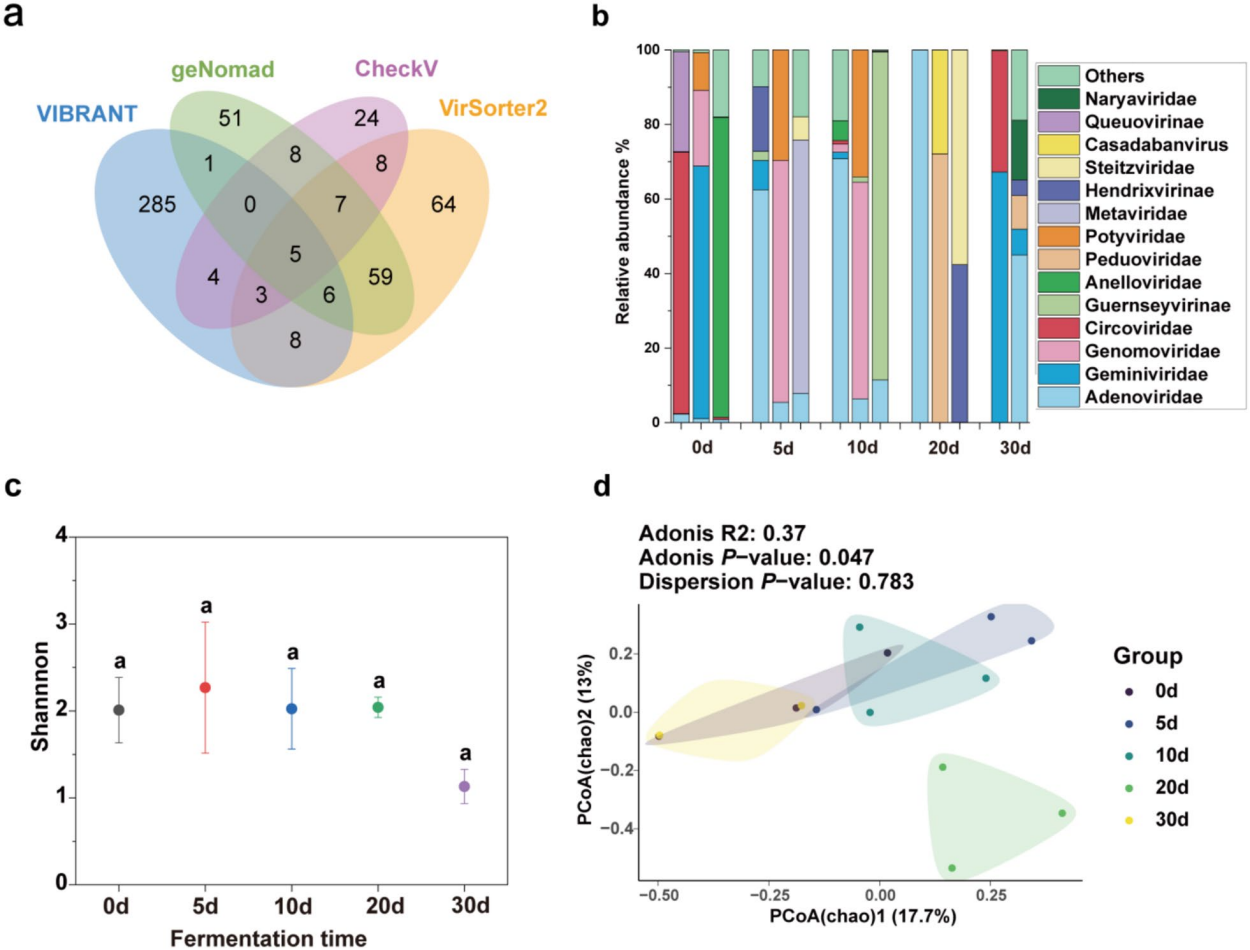
Profile of viral communities in fermented grains. **a** Identification of viral contigs larger than 2 kb using various tools in 14 fermented grains. **b** Relative abundance of the dominant viruses at family level (top 15) in fermented grains. **c** Viruses Shannon index, the same letter represents no significant difference. Data are presented as the means 3 standard deviations (by Tukey’s test, *P* > 0.05). **d** PCoA results of vOTUs based on Chao distance matrix, showing the distribution of samples at different time points (0 day, 5 days, 10 days, 20 days, 30 days). The sample from day 30 at 1.5 m failed to be sequenced due to issues with library construction

To evaluate the diversity and function of viral community in the fermentation of strong-flavor Baijiu, 534 viral contigs were obtained from 14 fermented grains (the sample from day 30 at 1.5 m failed to be sequenced due to issues with library construction) using 4 tools (Fig. 5a). VIBRANT identified the highest number of unique viral contigs (285), significantly exceeding the unique detections of the other tools. The largest overlap occurred between VirSorter2 and geNomad, with 59 shared predicted viral contigs. Only 5 viral sequences were detected by all four tools, underscoring the considerable differences in their viral detection capabilities. These results suggest that using multiple tools can enhance the comprehensiveness of viral contig predictions.

We clustered 534 viral contigs using ≥ 95% nucleotide identity and ≥ 85% coverage of the shorter sequence, choosing the longest contig as the representative. This resulted in a total of 513 vOTUs.

Since ICTV promulgated a new virus classification in 2021 (Zhang et al. 2024), we used the new virus classification to annotate the vOTUs. We found that the annotation rate of the new virus classification was low, with only 94 vOTUs being annotated at the family level (Fig. 5b). This indicates a large number of new, unannotated viruses in the Baijiu fermentation process. As fermentation progressed, the relative abundance of different viral families changed significantly. For instance, at day 0, the fermentation system was predominantly populated by viral families such as Circoviridae, Geminiviridae, and Anelloviridae. Although these families are primarily associated with plants, animals, and environmental reservoirs, their presence in the fermentation system suggests potential contamination from raw materials or the environment, as well as possible unexplored interactions with the microbial community (Bandoo et al. 2021). Their detection highlights the complexity of viral dynamics in fermentation ecosystems. Between days 5 and 10, the dominant viral families shifted to Adenoviridae, Genomoviridae, Metaviridae, and Guernseyvirinae, reflecting active viral–microbial interactions during the early-to-mid fermentation stages. Genomoviridae, a family of circular single-stranded DNA viruses, is often linked to bacterial and fungal associations, potentially influencing microbial gene exchange, genomic stability, or metabolic regulation (Malathi and Renuka Devi 2019). Metaviridae, a group of retrotranscribing viruses predominantly associated with fungi, may impact fungal metabolism and biomass turnover, thereby affecting fermentation efficiency and microbial succession (Llorens et al. 2020). Guernseyvirinae, a subfamily of phages, is likely involved in regulating bacterial populations by selectively infecting key fermentation-associated bacterial taxa (Lu et al. 2020). By day 20, the viral community composition transitioned to Adenoviridae, Peduoviridae, Hendrixvirinae, and Steitzviridae, suggesting a shift in viral influence on microbial succession. Peduoviridae, a lytic phage family known to infect Gram-negative bacteria, may play a crucial role in shaping bacterial populations by targeting ethanol- and acid-tolerant microbes such as *Gluconobacter* and *Acetobacter*, which are prevalent in fermentation environments (Turner et al. 2023). Hendrixvirinae, a subfamily of phages primarily infecting Firmicutes, could contribute to microbial community restructuring by modulating host populations and influencing fermentation-associated bacterial dynamics (de Jonge et al. 2024). Steitzviridae, a relatively understudied phage family, may help regulate microbial balance through host-specific interactions, although its exact role in fermentation remains to be fully elucidated (Valencia-Toxqui and Ramsey 2024). By day 30, Adenoviridae and Geminiviridae emerged as the most prevalent viral families, suggesting the persistence of viruses with possible environmental or microbial interactions. This dynamic shift in viral populations over time underscores the potential influence of phages and other viruses in shaping fermentation microbiota, which may have implications for fermentation efficiency, microbial stability, and metabolite production.

At the same time point, distinct differences in viral family composition were observed between samples. On day 5, the relative abundance of Geminiviridae was higher in the 0.5 and 1-m depth samples, whereas Metaviridae accounted for a larger proportion in the 1.5-m depth sample. Certain viral families were more prominent at specific depths. For example, in the 0.5-m depth samples on days 5, 10, and 20, Geminiviridae became the dominant viral family. Conversely, at other time points and depths, the relative abundance of this family was relatively low. These results illustrate the diverse composition of viral groups, including Potyviridae, Metaviridae, and Circoviridae. This highlights that the types of viruses in fermented grain samples are relatively abundant and dynamically change over time. Overall, the relative abundance and diversity of viral families varied over time, providing valuable data to support the study of viral dynamics at different stages.

We found that the alpha diversity of vOTUs remained relatively stable throughout the fermentation process (Fig. 5c). The PCoA results based on the Chao distance matrix illustrate the distribution of samples across different time points (0 days, 5 days, 10 days, 20 days, 30 days). Overall, the differences between these time-point groups are statistically significant (Adonis, *P* < 0.05), whereas the dispersion among samples within each group is not significant (Dispersion, *P* > 0.05) (Fig. 5d). These findings indicate that the time points have a significant impact on group differentiation, rather than on the variability within groups. The PCoA results based on Bray–Curtis distance indicated that there were no significant differences in vOTU abundance across different fermentation time points (Adonis, *P* > 0.05) (Fig S2), suggesting that the abundance of viral species remained consistent throughout the fermentation process. Additionally, PCoA analysis based on Bray–Curtis distance revealed no significant differences in vOTU abundance at varying depths (Adonis, *P* > 0.05) (Fig S3).

### Virus host prediction, functional genes, and their relationship with major microorganisms

**Fig. 6:**
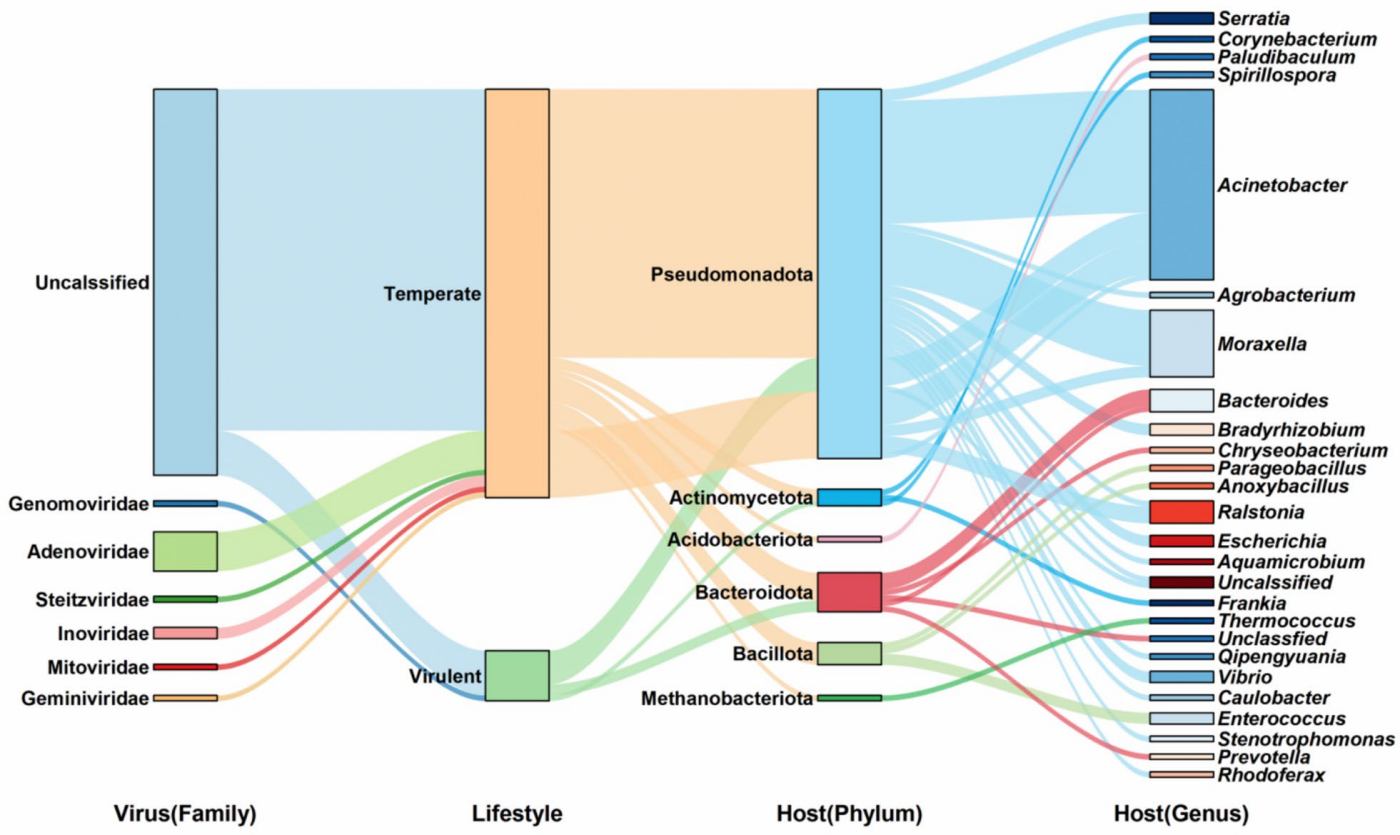
Viral host and lifestyle prediction. The relationship between viruses (at the family level) and bacteria (at the phylum and genus level) in the fermented grains. Different colored connecting lines show bacterial hosts (phylum and family level) and viruses. The thickness of the line represents the number of predicted hosts

Viruses, incapable of sustaining life activities independently, necessitate the reliance on compatible hosts (Chevallereau et al. 2022). We found 84 vOTUs (16.37%) that were able to predict the hosts at genus level based on iPhoP, where the most vOTUs (89.0%) were temperate lifestyles (Fig. 6). Of the viruses, 84.1% vOTUs were taxonomically assigned to uncalssified. Commonly predicted hosts included Pseudomonadota (80.4% of virus-host pairs) and Bacteroidota (8.5%). The top three predicted host genera were *Acinetobacter* (41.4%), *Moraxella* (14.6%), *Bacteroides* (4.8%).

To investigate the function of viral genes, we compared their predicted ORFs with the eggNOG database to obtain functional annotations. We found that viral gene functions were predominantly annotated for a large proportion of unknown functions (35.7%), followed by DNA replication, recombination, and repair (20.4%). In addition, we found that genes related to metabolism accounted for 13%. The metabolic pathways included carbohydrate transport and metabolism (4.2%), amino acid transport and metabolism (2.6%), inorganic ion transport and metabolism (1.9%), coenzyme transport and metabolism (1.4%), lipid transport and metabolism (1.3%), and secondary metabolite biosynthesis, transport, and catabolism (1.0%) (Fig S4). It is well known that viruses lack metabolic capacity and must rely on their hosts for life cycle activities (Sumbria et al. 2021). The genes responsible for these metabolic functions in viruses were acquired from the hosts and reintroduced to the host through infection, leading to changes in the host's metabolic capacity (Warwick-Dugdale et al. 2019).

**Fig. 7:**
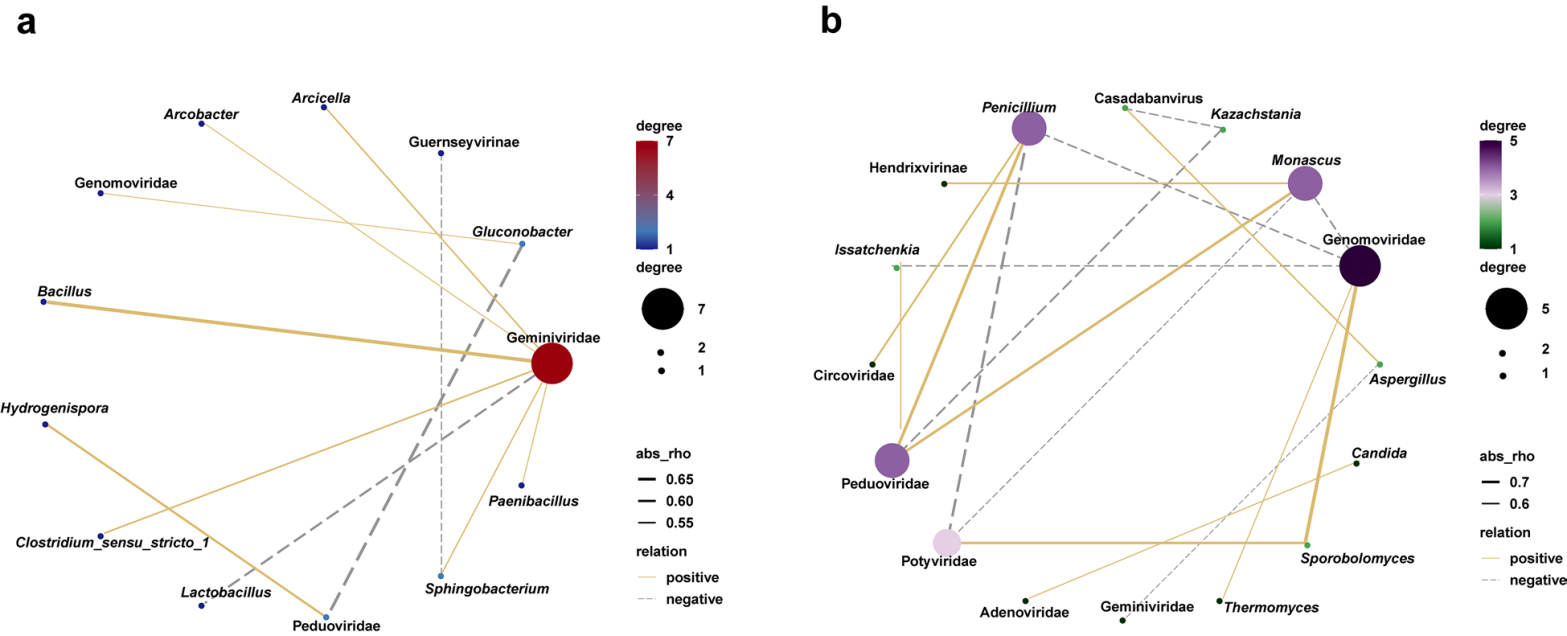
Spearman correlation of dominant viral families with dominant bacterial (**a**) and fungal genera (**b**)

To examine the correlations between dominant microbial genera and major viral families, Spearman’s rank correlation coefficients and statistical significance were calculated (Fig. 7). Geminiviridae showed positive correlations with *Bacillus*, *Clostridium_sensu_stricto_1*, *Arcicella*, *Arcobacter*, *Sphingobacterium*, and *Paenibacillus*, but was negatively correlated with *Lactobacillus*. Peduoviridae was negatively correlated with *Gluconobacter* (Fig. 7a), suggesting that Peduoviridae may directly lyse *Gluconobacter*. Notably, stronger correlations were observed between fungi and viruses. Genomoviridae exhibited positive correlations with *Sporobolomyces* but negative correlations with *Issatchenkia*, *Penicillium*, and *Monascus*. Genomoviridae is a relatively newly discovered family of single-stranded DNA (ssDNA) viruses that primarily infect fungi and plants (Malathi and Renuka Devi 2019). Peduoviridae was positively correlated with *Penicillium* and *Monascus*, yet negatively correlated with *Kazachstania*. As a family of phages, Peduoviridae does not directly infect these fungi but may indirectly influence fungal communities by modulating bacterial community structure, thereby affecting fungal dynamics (Turner et al. 2023). Potyviridae displayed a positive correlation with *Sporobolomyces* and a negative correlation with *Penicillium*. Meanwhile, Casadabanvirus was negatively correlated with *Kazachstania* but positively correlated with *Aspergillus *(Fig. 7b) *Aspergillus* is an important genus responsible for saccharase production during Baijiu fermentation, while *Kazachstania* contributes significantly to ethyl acetate production in strong-aroma Baijiu (Wu et al. 2021a, b; Yuan et al. 2023a, b). As a phage family, Casadabanvirus may influence fungal growth indirectly through virus-mediated horizontal gene transfer, potentially altering the metabolic pathways of host bacteria and leading to the production of metabolites that are detrimental to fungal development (Bi et al. 2022). These findings suggest a potential impact of viral families on the structure and dynamics of microbial communities. Although some viruses do not directly infect fungi or bacteria, they can influence microbial community composition through indirect mechanisms. These include selectively infecting and suppressing competing microbial taxa, reducing competition for ecological niches, and releasing nutrients via host cell lysis, thereby providing resources that support the growth of other microorganisms (Brown et al. 2022).

### Relationships between viruses, microorganisms and metabolites

**Fig. 8:**
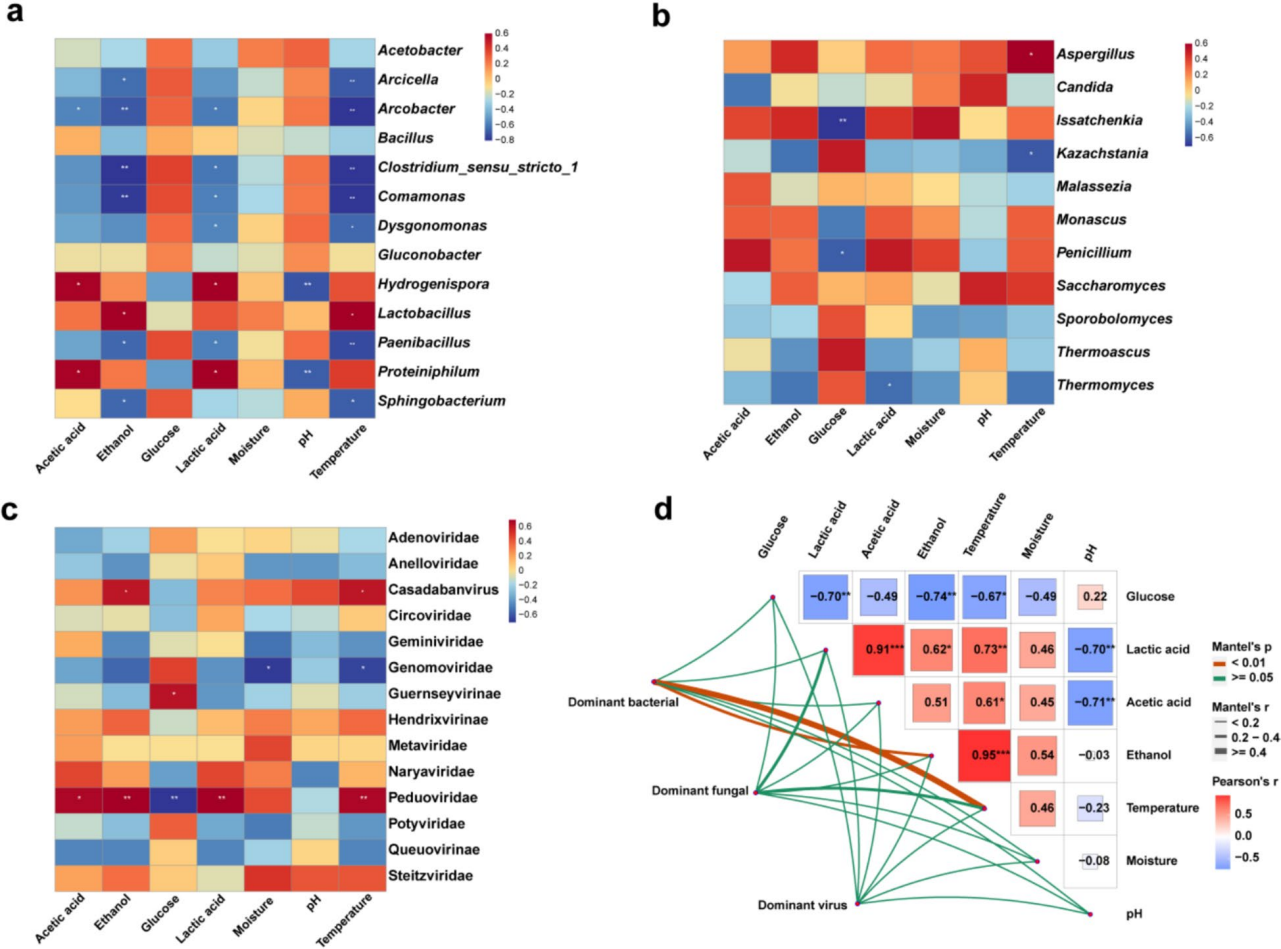
Correlation heatmaps of dominant bacterial (**a**), fungal genera (**b**), and viral families (**c**) with physicochemical factors, and Mantel tests linking microbial genera, viral families, and physicochemical factors (**d**). Dominant bacterial and fungal genera, viral family: relative abundance > 1%

The correlations between the dominant bacterial and fungal genera, viral family (relative abundance 1%) with physicochemical factors were calculated based on Spearman’s rank correlation (Fig. 8). As shown in Fig. 8a, *Lactobacillus* was positively correlated with ethanol and temperature. This genus exhibits high ethanol tolerance, allowing it to survive and proliferate in environments with elevated ethanol concentrations (He et al. 2021a, b). *Paenibacillus* was negatively correlated with lactic acid, ethanol and temperature. This genus is generally intolerant of acidic conditions, ethanol, and high temperatures, leading to a decline in its abundance under these environmental stresses (Grady et al. 2016). *Proteiniphilum* was negatively correlated with pH, while was positively correlated with acetic acid and lactic acid. As an anaerobic bacterium, *Proteiniphilum* thrives in low-pH environments. Acetic acid and lactic acid, as metabolic by-products, may serve as available carbon sources or alter the microbial community structure in ways that facilitate *Proteiniphilum* colonization (Liu et al. 2022). Notably, more significant correlations were found between fungi and physicochemical factors (Fig. 8b). *Aspergillus* exhibited positive correlations with temperature, as higher temperatures enhance its metabolic activity and promote the secretion of numerous extracellular enzymes (e.g., amylase, cellulase), enabling it to efficiently degrade complex carbon sources and adapt to the fermentation environment (Bellaouchi et al. 2021). *Issatchenkia* and *Penicillium* were negatively correlated with glucose. *Issatchenkia* rapidly metabolizes glucose in fermented grains to support its growth and produces flavor compounds such as ethanol and ethyl acetate (Guan et al. 2023). *Kazachstania* was negatively correlated with temperature. Elevated temperatures may disrupt its enzymatic activity or inhibit cell division, thereby limiting its growth (Mielecki et al. 2024). In addition, we calculated correlations between the major virus families and physicochemical factors (Fig. 8c). Genomoviridae was negatively correlated with moisture and temperature. Viral particles of this family may be less stable in high-temperature and high-humidity environments, leading to a reduced ability to infect. This instability may be related to the thermal sensitivity or structural fragility of its capsid proteins (Roos et al. 2007). Peduoviridae was negatively correlated with glucose, while was positively correlated with acetic acid, lactic acid, ethanol and temperature. High glucose concentrations typically promote rapid bacterial growth; however, this rapid proliferation may activate host defense mechanisms against phage infection (such as the CRISPR-Cas system or restriction-modification systems), thereby reducing the infection efficiency of Peduoviridae (Sun et al. 2023a, b). Furthermore, Peduoviridae may preferentially infect acid- and alcohol-tolerant bacterial hosts, such as *Gluconobacterium* or *Acetobacter*, which dominate fermentation environments rich in organic acids and ethanol (Philippe et al. 2018).

The Mantel test was performed to examine the correlations between community variation and physicochemical differences across dominant bacterial and fungal genera, as well as viral families. As shown in Fig. 8d, ethanol and temperature were significantly correlated with the dominant bacterial community, suggesting that these factors play crucial roles in shaping bacterial composition during fermentation. In contrast, most relationships between dominant fungal genera, viral families, and environmental factors were not statistically significant (represented by thin green lines), suggesting that these microbial groups may be more influenced by biotic interactions (e.g., competition, symbiosis, or host specificity) rather than direct environmental fluctuations.

Notably, a strong negative correlation was observed between glucose and ethanol (correlation coefficient: -0.74), which aligns with the typical metabolic conversion process during fermentation. Additionally, lactic acid and acetic acid exhibited a highly positive and statistically significant correlation, with a coefficient of 0.91. This suggests that the metabolic pathways governing lactic acid and acetic acid production are closely linked, possibly through bacterial cross-feeding or co-metabolism.

To investigate the correlations between dominant microbial genera and differential compounds, Spearman’s rank correlation coefficients and their statistical significance were calculated. A total of 83 significant positive correlations and 56 significant negative correlations were identified between 24 genera (13 bacterial and 11 fungal) and 38 flavor compounds (Fig S5). These findings suggest that variations in microbial communities contribute to differences in metabolic profiles. Notably, *Hydrogenispora* and *Proteiniphilum* showed positive correlations with the distribution of more than 17 compounds, highlighting their key roles in metabolite formation and transformation (Fig S5a). In contrast, *Gluconobacter*, *Dysgonomonas*, and *Arcobacter* were negatively correlated with more than 16 compounds. These bacteria may be particularly sensitive to various metabolic by-products, such as organic acids, alcohols, or esters, especially antimicrobial compounds that could disrupt their cell membrane integrity, enzyme activity, or metabolic homeostasis, ultimately leading to growth inhibition. Additionally, *Issatchenkia* exhibited a positive correlation with ethyl acetate, suggesting its involvement in ethyl acetate biosynthesis. This is likely attributed to its unique esterase/lipase system, which catalyzes the esterification of alcohols and acids, contributing to the production of this key flavor compound (Wang et al. 2022a, b). Meanwhile, *Thermomyces* and *Thermoascus* were negatively correlated with seven compounds (Fig S5b). As thermophilic fungi, they may be particularly sensitive to certain environmental compounds, such as antimicrobial organic acids, alcohols, or esters. The absence of efficient detoxification pathways could further limit their ability to tolerate these metabolites, thereby restricting their growth (Maheshwari et al. 2000).

## Discussion

Understanding the succession of microorganisms during food fermentation is crucial for producing high-quality products (Paillet et al. 2024). Viruses may influence this succession (Zhang et al. 2024); however, limited information exists regarding their dynamics in fermented grains. To the best of our knowledge, this study represents the first investigation into viral diversity during the fermentation of strong-flavor Baijiu. We described the changes in viral and microbial communities throughout the 0–30 day fermentation period of strong-flavor Baijiu, and calculated the relationship between viruses, microorganisms, and flavors. This study uncovered a potential association between viral families and microbial genera using Spearman rank correlation analysis. The dominant phage families identified were Peduoviridae, Hendrixvirinae, Steitzviridae, Casadabanvirus, and Queuovirinae. Notably, Peduoviridae showed a positive correlation with *Penicillium*and *Monascus*, but a negative correlation with *Kazachstania* and *Gluconobacter*. These findings offer a new perspective for understanding the mechanisms underlying the dynamic changes in microbial communities during liquor fermentation. They suggest that viruses may play a significant role in regulating the structure and function of microbial communities, contributing to the advancement of microbial community ecology theory.

Baijiu fermentation is a complex multi-strain mixed fermentation process (Wang et al. 2024), where traditional culturable methods have limited throughput. In contrast, genomic sequencing provides a more comprehensive approach to capturing the succession dynamics of microbial and viral communities (Zhang et al. 2024). In this study, we employed a combination of advanced viral identification tools, implemented rigorous quality control measures, and adhered to the latest viral classification standards. Compared with previous virome studies on cheese (Paillet et al. 2024), cider (Ledormand et al. 2022), and vinegar (Ma et al. 2024), our findings reveal that the majority of viral hosts in the Baijiu fermentation system are non-fermentative functional bacteria. This suggests that viral activity may play a crucial role in maintaining microbial balance, potentially facilitating a smoother and more stable fermentation process. In addition, this study addresses a critical gap in research on viral diversity in Baijiu fermentation, enhances our understanding of viral communities within the fermentation ecosystem, and provides essential foundational data for future studies.

In previous studies, we used virome technology to explore the viral community during sauce-flavor Baijiu fermentation (Du et al. 2022). However, the viral sequence identification method used at that time relied on database comparison, which is no longer the mainstream approach for viral identification and may lead to the omission of certain viral sequences (Camargo et al. 2023). Additionally, comparison-based methods can result in false positives due to the absence of marker genes in some viruses (Ren et al. 2017). Furthermore, the viral classification criteria used then are no longer applicable (Zhu et al. 2023), and host information was also absent. While electron microscopy was used to observe viral particles, a wet-lab test was conducted to confirm that the phage inducer inhibited the growth of *Bacillus licheniformis*. Moving forward, we plan to reanalyze both datasets using the latest virus identification and classification tools to compare the differences between the two aroma viral communities.

In addition to the phage families that infect bacteria, we identified several families such as Adenoviridae, Geminiviridae, and Retroviridae (Fig. 5b), which primarily infect eukaryotes or other organisms rather than bacteria (Benkő et al. 2022; Coffin et al. 2021; Zerbini et al. 2017). This presence can be attributed to the open fermentation environment of Baijiu production (Wu et al. 2021a, b), where raw materials are exposed to the surroundings, allowing these viruses to enter the fermentation system alongside the ingredients. Generally, these viruses do not pose a threat to the safety of the Baijiu, as their infectivity or activity within the fermentation process has not been experimentally verified. Moreover, even if they are active, they are likely inactivated during the distillation process required for producing drinking liquor. Additionally, while these viruses are present, they do not significantly impact the ethanol and main flavor compounds during fermentation (Figs. 1 and 2). Research on the isolation, culture, and activity verification of these viruses in Baijiu fermentation remains scarce.

We also faced one of the major challenges in viral metagenomics: a low proportion of viral sequences with host predictions. Although we utilized iPHoP (Arumugam et al. 2023) for host prediction—an integrated tool that combines multiple methods and has demonstrated superiority over individual approaches—we were surprised not to detect LAB phages (Fig. 6). We employed various tools for viral sequence identification and implemented strict controls (Fig. 5a), leading us to speculate that the initial viral particle enrichment process may have inadvertently filtered out LAB phages. Optimization of viral particle enrichment methods may be necessary, similar to the approaches taken in cheese and cider studies (Dugat-Bony et al. 2020; Ledormand et al. 2022). These could involve refining various steps, from selecting the appropriate buffer to choosing the most effective enrichment techniques, such as tangential flow filtration (TFF), precipitation, or ultracentrifugation (Zhang et al. 2024). Additionally, optimizing viral DNA extraction methods, including the use of phenol–chloroform extraction or selecting the most suitable extraction kits, could further enhance recovery and analysis. In addition, we performed multiple displacement amplification (MDA) method for viral genome amplification in this study. However, this method may introduce amplification bias (Parras-Moltó et al. 2018). Achieving sufficient viral DNA yields would eliminate the necessity for the whole-genome amplification step. Hence, we recommend optimizing the viral enrichment and DNA extraction processes prior to conducting virome sequencing on your unique samples. Alternatively, combining two sequencing methods can provide a more comprehensive view of the viral community. Previous studies indicated that metagenomics sequencing tends to perform better than virome sequencing in acidic soil environments (Bi et al. 2024). Different environmental sample metagenomics and viromes could obtain different viral community characteristics (Kosmopoulos et al. 2024). Therefore, using both sequencing methods concurrently on fermented grains may help address this issue.

During the viral gene annotation process, we identified several genes with metabolic functions (Fig. 7), such as carbohydrate, amino acid, inorganic ion, coenzyme, lipid, and secondary metabolite biosynthesis, transport, and catabolism, which may represent the potential added value of bacterial infection by phages. Although viruses lack intrinsic metabolic capabilities, their genes may influence host metabolism through infection (Sun et al. 2023a, b). This insight offers valuable clues for investigating the potential mechanisms by which viruses impact the Baijiu fermentation process, thereby deepening our theoretical understanding of their role in fermentation systems. While this finding is intriguing, dedicated experiments should be designed to investigate this further—such as assessing the fitness of specific strains in a defined medium lacking essential compounds, in the presence or absence of a phage carrying genes involved in the biosynthesis of those compounds.

In summary, we observed dynamic changes in microorganisms and the virome, alongside physicochemical and flavor compounds, during the fermentation of strong-flavor Baijiu. The microorganisms exhibited significant successional changes, while major viral families persisted throughout the fermentation process. This study employed Spearman correlation analysis to explore potential associations between viral families and microbial genera. These findings offer a novel perspective on the dynamic mechanisms shaping microbial communities during Baijiu fermentation, suggesting that viruses may play a crucial role in regulating microbial community structure and function. These insights contribute to the advancement of microbial community ecology theory and deepen our understanding of microbial interactions in fermentation processes.

Gaining insights into viral community ecology is essential for a comprehensive understanding of microbial succession during Baijiu fermentation.

## Supplementary Information

Supplementary material 1.

## Data Availability

The datasets used during the current study are available from the corresponding author on reasonable request.
