# Supplementary material for: Unraveling the multiple interactions between phages, microbes and flavor in the fermentation of strong-flavor Baijiu
Source: Bioresour Bioprocess. 2025 Mar 5;12(1):14. doi: 10.1186/s40643-025-00852-1 (PMC11883080; doi:10.1186/s40643-025-00852-1)
Supplement: Supplementary file 1 — Supplementary material 1. [file 40643_2025_852_MOESM1_ESM.docx]

**
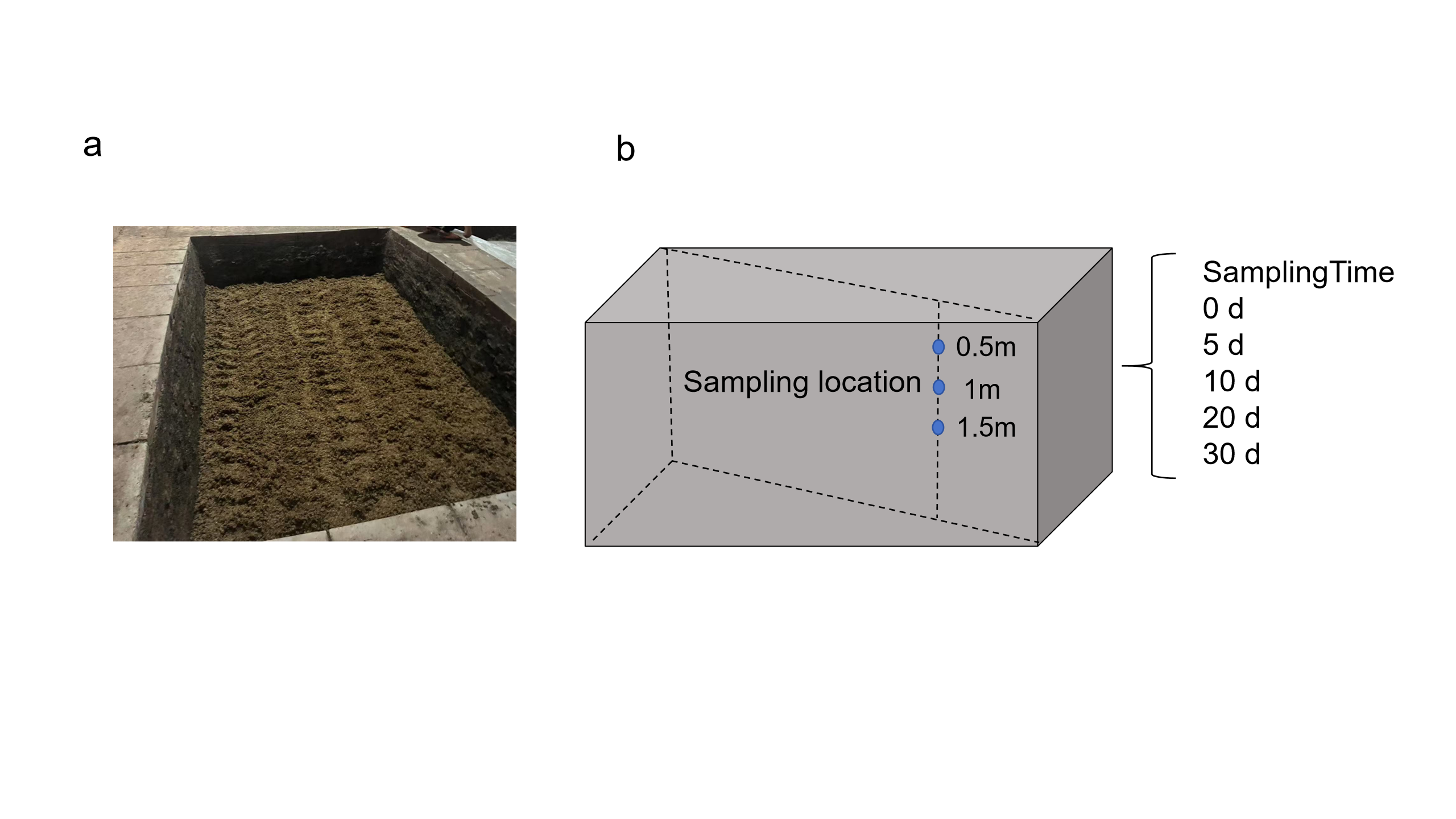
**

**Fig S1 Sampling information for fermented grains of strong-flavor Baijiu**. (a) Fermentation environment scene. (b) Blue spots represented the sampling point. The sampling times are days 0, 5, 10, 20, and 30, respectively.


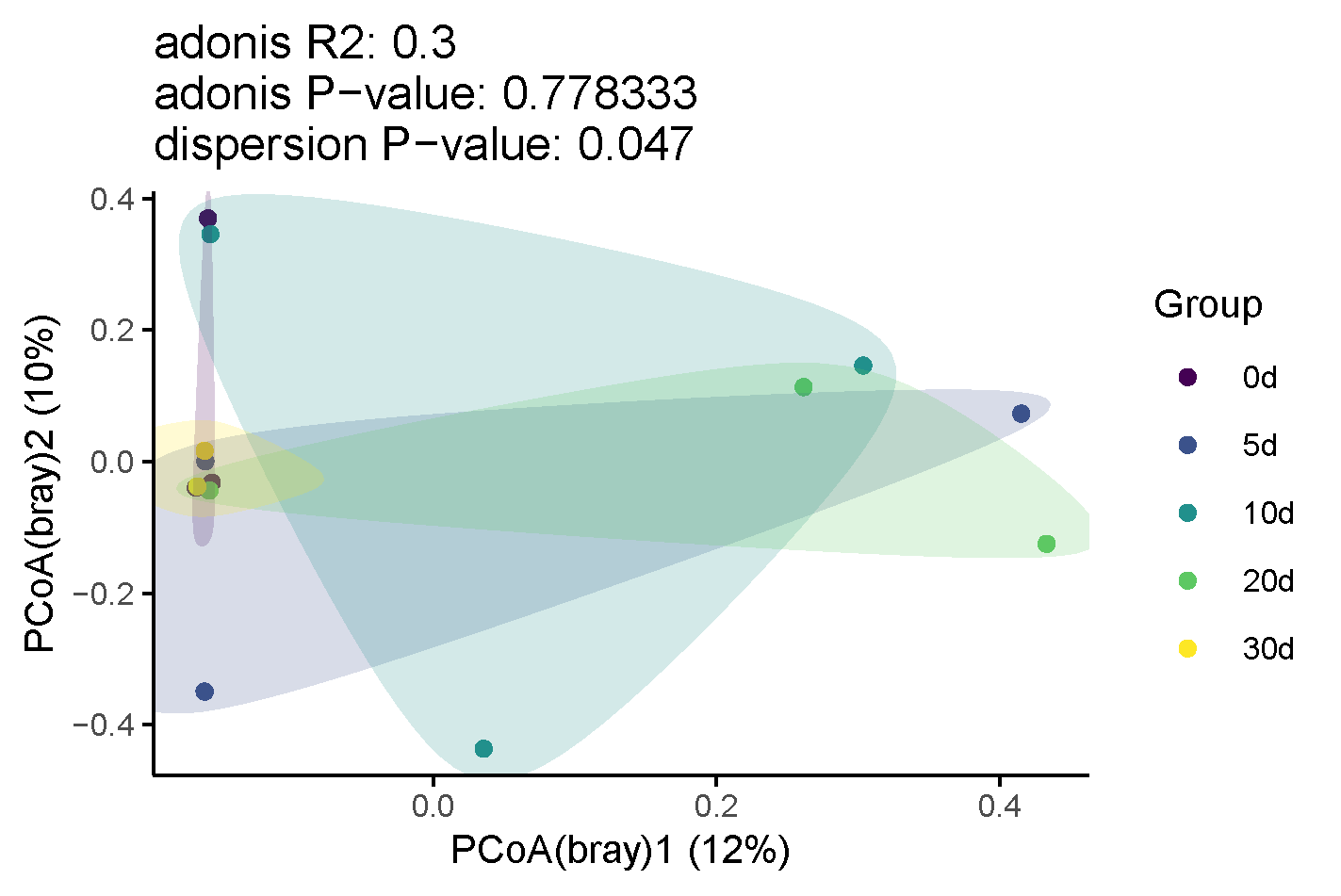


**Fig S2 PCoA results of vOTUs based on Bray-Curtis distance matrix at different time points.**

**
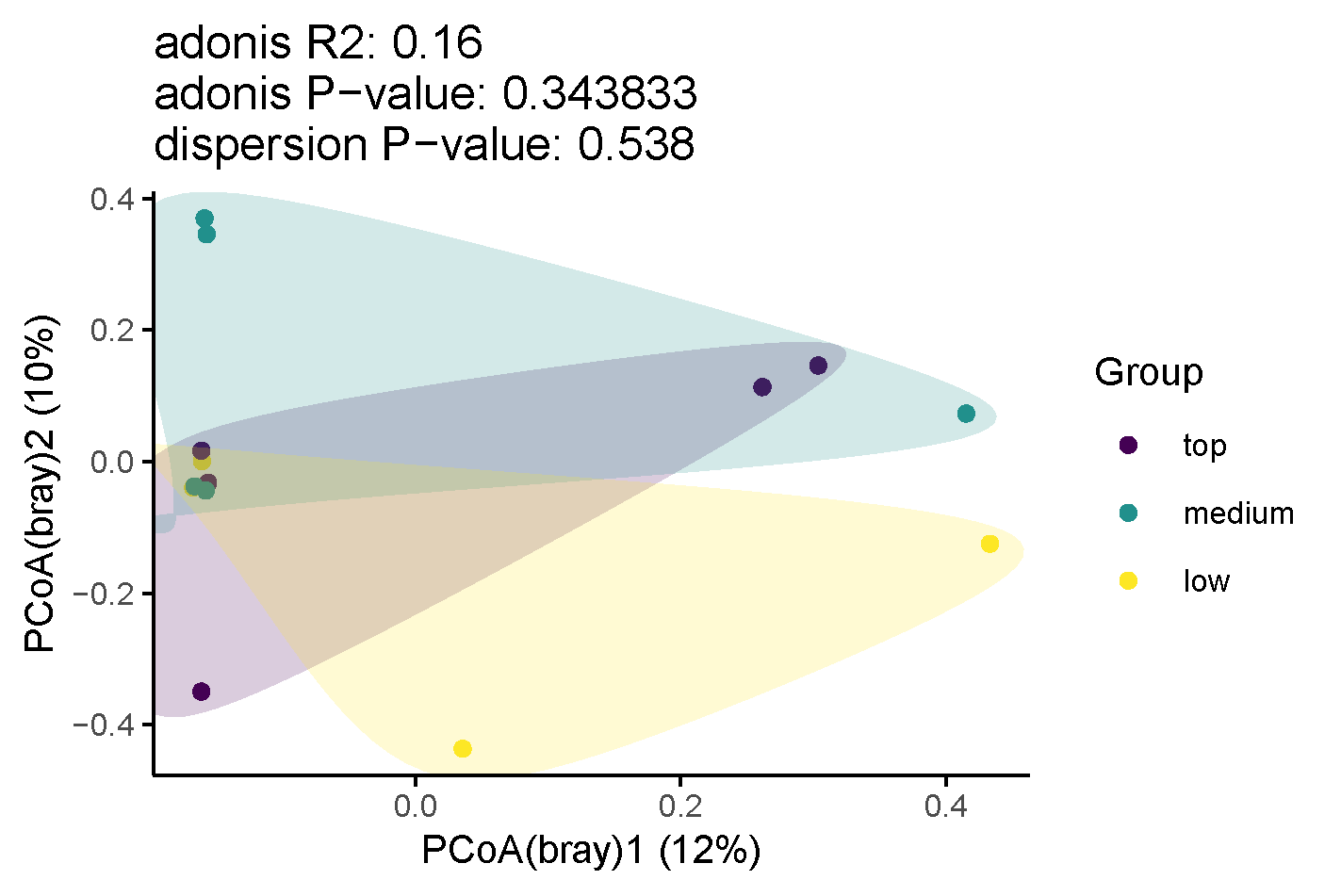
**

**Fig S3** **PCoA results of vOTUs based on Bray-Curtis distance matrix at different depths.**


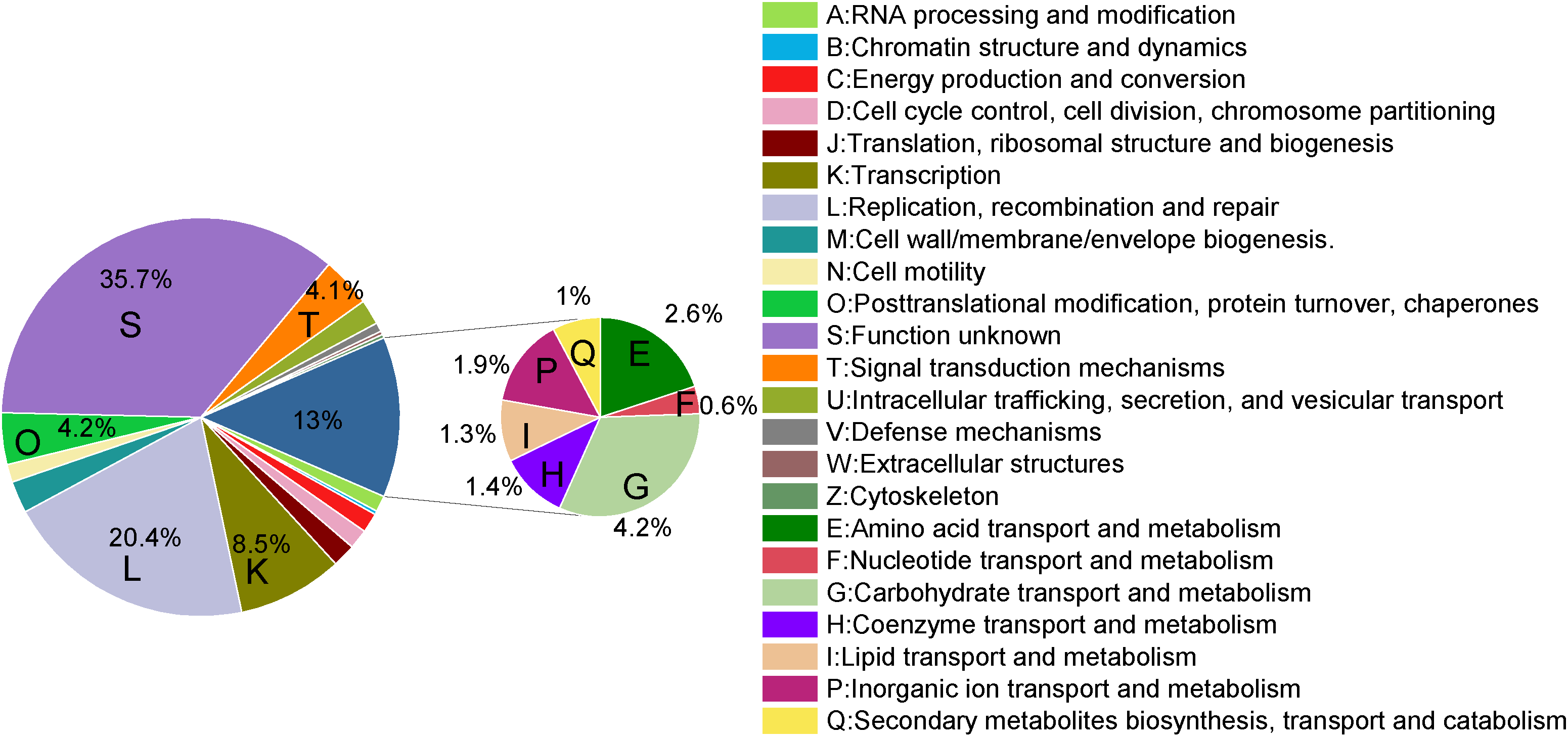


**Fig S4 Functional gene annotation of viruses in fermented grains.** Viral gene functions annotated by the COG database.


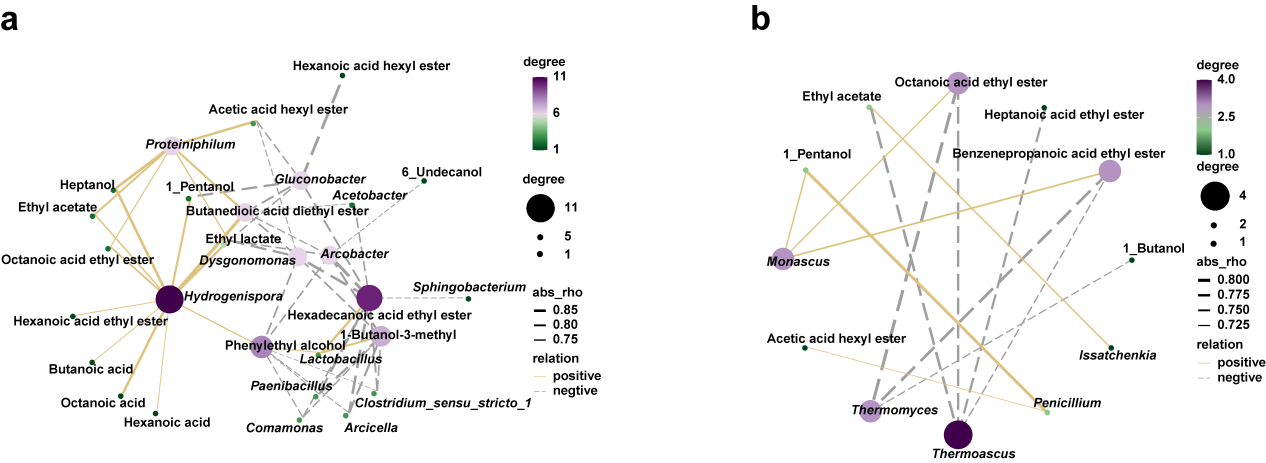


**Fig S5** **Correlation network between dominant microorganisms and differential compounds based on Spearman correlation rank test (*p* < 0.05 and |ρ| ≥ 0.7).** Dominant bacterial (a) and fungal genera (b).
